# An Examination of Health Care Transition Experiences Through Parents’ Reflections About Their Sons or Daughters Who Have Intellectual and Developmental Disabilities

**DOI:** 10.3390/children12070886

**Published:** 2025-07-04

**Authors:** Christine B. Mirzaian, Rowan Smith, Cecily L. Betz

**Affiliations:** 1 Department of Pediatrics, University of Southern California Keck School of Medicine, 4650 Sunset Blvd. Mailstop 53, Los Angeles, CA 90027, USA; cmirzaian@chla.usc.edu; 2 University Center for Excellence in Developmental Disabilities, Children’s Hospital Los Angeles, 4650 Sunset Blvd. Mailstop 53, Los Angeles, CA 90027, USA; rosmith@chla.usc.edu

**Keywords:** health care transition, transfer of care, youth and young adults, intellectual and development disability

## Abstract

Background: As national and international reports reveal, significant health care transition (HCT) service disparities exist for youth and young adults with intellectual and developmental disabilities (YYAs with IDD). The development of the HCT model necessitates informed perspectives from a broad constituency, including consumers and families. Parents’ retrospective perspectives of their sons’ or daughters’ HCT experiences are presented to enlarge the understanding of the service need. Methodology: Eleven parents were recruited virtually from parent support/disability advocate groups via an email distribution list of the Children’s Hospital Los Angeles University Center for Excellence in Developmental Disabilities. Parents who consented to participate were interviewed by phone using an interview guide with 11 open-ended items. Three questions focused on the barriers and facilitators associated with the HCT experience are reported. Findings: Four major themes were generated from the analysis of data gathered from parents pertaining to their sons’ or daughters’ health care transition experiences, focusing on the transfer of care. Two major themes were related to HCT barriers—Pediatric Care Contrasted with Adult-Focused Care and Transfer of Care Barriers—and two were related to HCT facilitators—Transfer of Care Facilitators and Transfer of Care Recommendations. Each of the major themes included subthemes. Conclusions: Parents openly shared their sons’ or daughters’ HCT experiences, which illuminated the scope of their challenges and the assistance received. These insights provide rich descriptions of the barriers they and their adult children faced as they proceeded with navigating new systems of health care. The reported data find support in other previously conducted studies.

## 1. Introduction

Improvements in the survival rates of youth and young adults with childhood-acquired illnesses and disabilities have created a widespread need of the service system to develop and implement linkage services between pediatric and adult-focused systems of health care, widely known as health care transition (HCT) services [1,2,3,4,5,6,7]. It is now estimated that 1.2 million young adults with chronic conditions or functional limitations enter into adulthood annually and thereby engage in the forthcoming transfer of care process that begins, depending on local and state guidelines, at 18 years of age [8]. Currently, extensive national and international efforts are underway to create formalized service bridges between these two disparate service systems, as no integrative infrastructure exists, even in countries with national health care. Recognizing the importance of addressing this unmet large-scale service need, service model recommendations have been generated by health care leaders and policymakers to facilitate HCT service models’ development and implementation [9,10,11,12,13].

These collective efforts have advanced the development of evidence that has facilitated HCT practice and research. Although promising growth has been made, much work needs to be accomplished. The implementation of HCT models reported in the literature could be described as promising, but it focuses prominently on selected chronic conditions, such as cystic fibrosis, sickle cell disease, and chronic kidney disease, due to HCT condition-focused initiatives by specialty organizations and HCT-focused researchers [9,10,11,12,13,14,15,16,17]. However, more universal HCT models that cross medical conditions are lacking.

It is evident that with the growing proliferation of models of care, those for individuals with IDD are lagging. It is well documented that HCT service disparities exist for the IDD population [18,19,20]. It is both relevant and timely to investigate the needs of youth and young adults in HCT services to gain a better understanding of service models to promote successful HCT outcomes for this community.

In addition, there is growing body of literature indicating that a major barrier to successful HCT for individuals with disabilities is finding adult-serving medical providers willing to care for them, as physicians themselves admit to showing bias against individuals with disabilities in qualitative studies [21,22,23,24], and surveys indicate that physicians’ knowledge of obligations to treat patients according to the legal obligations stated in the American with Disabilities Act (ADA) is poor [25]. While evidence of this bias exists in the literature from the providers’ perspective, it has not been well documented from the perspective of parents whose children have experienced the process of HCT from pediatric to adult-focused care.

Asking the parents of youth and young adults (YYAs) who have transitioned to adult-focused care and adulthood provides unique perspectives to assist service providers in the development and implementation of HCT service models to facilitate improved HCT outcomes. The purpose of this study was to explore parental retrospective perspectives about their sons’ or daughters’ HCT experience.

## 2. Methods

This study was approved by our Institutional Review Board (IRB) (approval #CHLA-22-0027, date of approval, 10 October 2022). Consent was not obtained from parents, as this study was considered exempt due to no personal identifying information being gathered. An information sheet was distributed with recruitment flyers. Parents who responded to the recruitment emails and indicated their willingness to participate were considered to have given their consent to participate in the study. Data were gathered using an 11-item interview guide composed of open-ended questions about parents’ experience with their young adult child’s HCT. Parents were invited to participate via an email with a recruitment flyer sent to the listserv of our University Center for Excellence in Developmental Disabilities (UCEDD), which includes approximately 3000 email addresses. The flyer was also shared among community-based organizations that focus on care for individuals with IDD. Initially, the research team received more than 160 emails, some of which were determined to be bots (i.e., bots are automated emails that are not purposeful responses to original email queries). Apparently, one of the email recipients had forwarded the email to a social media platform without the knowledge of the research team. After the emails were screened and the bots were removed, 60 individuals (approximately 2% of those initially emailed) indicated their interest in participating in the study. All 60 individuals were emailed to elicit their ongoing interest in participating in the study. Of this group, some individuals were excluded for the following reasons: they wanted to take part in Zoom interviews instead of phone interviews, did not have a child with a disability, or did not want to speak on the phone and wished to participate via email only. Eighteen individuals were scheduled for an interview, but of those, only ten (55% of those originally scheduled) participated on the day of their scheduled interview. An additional parent directly requested to participate in the study, as information was provided during one of the UCEDD Community Advisory Committee meetings. Recruitment occurred over a 1-month period from 24 May 2023 to 22 June 2023.

Parents who were interested contacted our research team and were screened for inclusion criteria, which included the following: (a) they identify as parents/legal guardians of an individual with an intellectual or developmental disability who has transitioned from pediatric to adult health care; (b) they are parents/legal guardians who are adults (≥18 years old); (c) they are parents/legal guardians who are English speaking, non-English speaking or with limited English proficiency; (d) they are parents/legal guardians who are pregnant. Exclusion criteria for this study were as follows: (a) parents/legal guardians who are unable to consent; (b) parents/legal guardians who are not yet adults (infants, children, or teenagers); (c) parents/legal guardians who are prisoners. Parents who agreed to take part in this study then participated in semi-structured interviews.

### 2.1. Interview Guide

The interview guide was composed of 11 open-ended questions. The entire interview focused on four areas of health care transition affecting individuals with IDD and their families: (a) barriers encountered and support and resources that facilitated their children’s transfer of care to adult-focused health care; (b) barriers encountered and support and resources that facilitated transition to postsecondary programs for education and employment and housing and community-based programs; (c) parental advice to other parents who will engage in future health care transition planning; and (d) input on the feasibility and benefits of a peer navigator program, including their children’s potential involvement as a recipient or provider of services [26].

These questions were generated based on the literature exploring health care transition issues involving the IDD population and the expertise and expertise in IDD and HCT of the research team [5,17,27]. The final selection of interview items was reached by team consensus. For the purposes of this investigation, the three questions in the interview guide focused on the barriers and facilitators associated with the transfer of care to adult-focused care are reported here. The questionnaire items used in this study are reported in Box 1. Data were collected anonymously to protect the identity of participants, so they could speak openly about issues with service provision without any concerns of being identified.

Interviews were conducted over the phone in either English or Spanish (if in Spanish, a certified interpreter with training in medical interpretation was utilized) by one of the research team members (R.S.) who had studied to a Master’s level and had previous research experience. Interviews lasted approximately 30 min each. Participants were provided with a USD 50 gift card incentive after the interview was completed. Parents were not asked to review the transcripts for accuracy, as the recordings and transcripts were verified records of the interviews.

Interviews were conducted until thematic saturation was reached. At the initiation of the study, it was anticipated that 10–20 interviews would be needed to reach thematic saturation. The research team (C.M. and C.B.), in consultation with the research team member who conducted the interviews (R.S.), judged that thematic saturation was achieved when no significant new insights were reported by the eleven parents who were interviewed.

Box 1 **Study Interview Questions.**

1. Do you feel as though you had the necessary resources to successfully transition your son/daughter to adult health/mental health care providers?
2. What advice would you offer to parents whose son/daughter have transferred their care to adult health/mental health care providers?
3. What did you find helpful or not helpful during your son’s/daughter’s transfer of care to adult health/mental health care providers?

### 2.2. Demographic Data

Eleven parents of sons or daughters who had completed the HCT experience and the transition to adulthood participated in this study. Data were gathered anonymously, so scant demographic data were gathered (Table 1). Of the eleven parents, two were parents of daughters. The IDD diagnosis of sons and daughters was revealed by six parents. There were two sons with autism; two sons with Down Syndrome; one son with Epilepsy; and a daughter with autism. All parents who participated in the study were mothers. One interview was conducted in Spanish.

### 2.3. Analysis of Interview Data

The interview recordings were transcribed and uploaded into NVivo qualitative analysis software [28]. The authors (CB, CM, and RS) then used the Framework Method of Qualitative Analysis [29] to analyze the data, following the steps detailed in Box 2. Initially, all three members of the study team (CB, CM, and RS) each coded the interviews independently. They then met multiple times to discuss their codes and resolve differences. A new codebook with agreed-upon codes was created, and then CB and CM re-coded all of the data using the new codebook. At that time, codes were grouped into larger themes by group consensus, with subthemes falling under each major theme.

Box 2 **Framework Method of Qualitative Analysis.**

Interpretation Process Used

Stage 1: Transcription. Described above.

Stage 2: Familiarization with the interview. All members of the study team read the interview transcripts.

Stage 3: Coding. After familiarization, as above, each member of the study team (RS, CM, and CB) conducted initial coding of the interviews. The team then met to discuss the codes and resolved discrepancies to work toward a final codebook.

Stage 4: Developing a working analytical framework. After a final codebook was created, the study team met again and discussed how the codes could be grouped into larger concepts (or “themes”), which created the basis for a working analytical framework.

Stage 5: Applying the analytical framework. After deciding on larger themes, the codes were reorganized into larger themes (see Table 2, Codebook).

Stage 6: Charting data into a framework matrix. Based on the larger themes, tables organizing the data were created.

Stage 7: Interpreting the data. Based on the created tables, general trends and conclusions were formulated.

**Table 2:** Codebook.

Barriers
A: Barrier_Access to Services_Need to Know to Ask for Services
B: Barrier_Adult Care Not the Same as Pediatric Care
C: Barrier_Adult Therapy Not the Same as Pediatric Therapy
D: Barrier_Day Program_Do Not Know What Programs Are Available
E: Barrier_Day Program_Lack of Staffing
F: Barrier_Day Program_Not Appropriate
G: Barrier_Day Program_Waitlist
H: Barrier_Education_Do Not Know About Self-Determination
I: Barrier_Education_No Transition Support Provided
J: Barrier_Education_Transition Support Not Adequate
K: Barrier_Education_Vocational Training Inadequate
L: Barrier_Employment_Loss of Benefits
M: Barrier_Employment_Transition Support Not Provided
N: Barrier_Independent Living_Lack of Resources
O: Barrier_Need for Adult Supervision
P: Barrier_Challenges Pertaining to Conservatorship
Q: Barrier_Parents Expected to Complete Transition Alone
R: Barrier_Post Secondary Education_Loss of Previous Aide
S: Barrier_Public Benefits Take a Long Time
T: Barrier_Self Determination_Denials Based on Self Determination
U: Barrier_Self Determination_Long Process and Lose Staff
V: Barrier_Self Determination_Services Still Inadequate
W: Barrier_Transfer of Care Difficulty in Finding Provider_Waitlists
X: Barrier_Transfer of Care_Changes in Insurance
Y: Barrier_Transfer of Care_Communication with Medical Providers
Z: Barrier_Transfer of Care_Difficulty Finding Providers (Insurance, Level of Comfort)
AA: Barrier_Transfer of Care_Difficulty Finding Provider_Mental Health
AB: Barrier_Transfer of Care_Difficulty Finding Provider_Primary
AC: Barrier_Transfer of Care_Difficulty Finding Providers_Specialists
AD: Barrier_Transfer of Care_Difficulty in Finding Provider_Dentist
AE: Barrier_Transfer of Care_Difficulty Obtaining Medical Supplies or Equipment
AF: Barrier_Transfer of Care_Provider Not Experienced with Disability
AG: Barrier_Transfer of Care_Providers Do Not Talk To Each Other
AH: Barrier_Transfer of Care_Providers Do Not Understand Conservatorship
AI: Barrier_Transfer of Care_Sudden Changes in Insurance
AJ: Barrier_Transfer of Care_Sudden Need to Change Doctors
AK: Barrier_Parents Feel Employment Not an Option
AL: Barrier_Transportation_No Transportation Available
Facilitators
A: Facilitator_Child is Able to Use Transportation Independently
B: Facilitator_Child Practicing Self Advocacy
C: Facilitator_Education_Transition Support Provided
D: Facilitator_Employment_Transition Supports Adequate
E: Facilitator_Familial Support
F: Facilitator_Finding Med Peds Doctor
G: Facilitator_Having Friends as Support
H: Facilitator_Listening to Parent
I: Facilitator_Parent Networking
Parent Concerns
A: Parent Concern_Community_Parents Age
B: Parent Concern_Insurance Coverage
C: Parent Concern_Loss of Benefits
Parental Belief
A: Parental Belief_Adult Child Not Employable
B: Parental Belief_Community Living Not Possible
C: Parental Belief_Feel Pushed Out of Process
D: Parental Belief_Independent Living_Adult Child Cannot Live Independently
E: Parental Belief_Postsecondary Education Not Possible
F: Parental Belief_Transition is Difficult
Transfer of Care Recommendations
A: Transfer of Care Recommendation_Know Appropriate Age of Transition
B: Transfer of Care Recommendation_Pediatrician Refer to Adult PCP
C: Transfer of Care Recommendation_Start Early_Switch PCP Early

## 3. Results

Four major themes were generated from the analysis of data gathered from parents pertaining to their sons’ or daughters’ health care transition experience, which focused on the transfer of care. Two major themes were related to HCT barriers: Pediatric Care Contrasted with Adult-Focused Care and Transfer of Care Barriers. Two of the major themes pertained to HCT facilitators: Transfer of Care Facilitators and Transfer of Care Recommendations. Each of the major themes consists of several subthemes, which are presented below.

### 3.1. Theme One: Pediatric Care Contrasted with Adult-Focused Care

Several parents identified the differences they experienced as barriers when their sons’ or daughters’ care was transferred from pediatric to adult-focused care, as described in this theme. As the parents described the challenges, it was apparent that they had not been forewarned by their pediatric team of the forthcoming changes they would encounter. Similarly, if some guidance had been provided, it would have been insufficient to assist them with navigating this new system of care. Their navigation skills and knowledge of the pediatric system of care did not fully translate as easily applicable to the adult-focused system of care. This realization was unnerving and distressing for parents as they were no longer confident in accessing services as they once were in the pediatric system of care.

These new challenges were evident as they spoke about the differences in the service models, as exemplified by the following two subthemes—Adult Care Not the Same as Pediatric Care/Adult Therapy Not the Same as Pediatric Therapy and Parents Expected to Complete Transition Alone—described below.

#### 3.1.1. Subtheme One: Adult Care/Therapies Not the Same as Pediatric Care/Therapies

Several parents who were interviewed shared their experiences of the distinctive changes they noted as their sons’ or daughters’ care was transferred to adult-focused providers. They noted differences between the health care models and available therapies, which are described as follows: Several parents raised issues related to the contrasting differences between pediatric and adult systems of care that they experienced as their children transitioned to adult-focused care. One parent (Parent 9) noted the lack of coordination of services in adult services:

“*Anything GI-related, formula, it was all one person. Now it’s like, you go see the GI, they say, oh, I don’t do that, the nutritionist has to do that. You have to see multiple doctors.*”

Another parent (Parent 8) reacted with the following observation: “*But for so many years, we had the same people. The same eye doctors and dentists, and pediatrician. And we were all set, you know? And then all of a sudden it was like, God, you know, this struggle to get to that point right now. And it’s still a struggle. But it just seems like a lot of people that, you know, especially with Down syndrome, they don’t know what to do.*”

Parents revealed their recognition of the evident differences between pediatric and adult-focused therapies. These service differences were characterized as no longer available in the adult system of care. One parent (Parent 11) noted the differences with the availability of services as an adult compared to pediatric care, with the following comments: “*He cannot get the services because the speech therapy, once they are adults, it’s not the regular speech therapy. It’s a speech therapy that’s specialized to give speech therapy to adults. And that, there is very few because of the insurance. The insurance has that, so he’s not getting services because at the location he was referred, there is just one speech therapy that sees adults. And this person is full booked up for months, I think eight months.*”

Although another parent (Parent 9) eventually located the services for her daughter, she acknowledged the differences between the service models. Her daughter’s services were now focused on divergent treatment goals in contrast to what she received previously and were time-limited: “*And so, when I finally found a place, and there’s a waiting list. And even when she got in there, the model is for rehab, as opposed to therapy, which means they’re working toward when they can kick you out, [LAUGHS] for whatever reason. Just pick one.*”

#### 3.1.2. Subtheme Two: Parents Expected to Complete Transition Alone

Multiple parents expressed feeling alone in the transition process or not having adequate support. One of the parents (Parent 4) expressed her feelings of facing service challenges alone. She felt she could not find the support needed to assist her with obtaining services for her daughter, who has mental health needs. She described her service dilemma as creating unwanted pressure: “*I would also point out again that the process with kids with mental health issues or disabilities, they put too much pressure in the parents. There is not support for the parents. Also you have to take in account that there are families around that special kid and that kid with disability and mental health kid. And they receive all of the weight and they also need support for their mental health. And it is, you know, emotional up and down each one of the relatives in the family. And we get too much pressure over us. There are many things. And I think that they have to solve that. If you’re a professional and you get paid, you should give the solutions. And they are just putting too much pressure over the parents. And I think that would be something important*.”

Another parent (Parent 10) stated, “*And then the moment, it’s literally like the clock turns to their, you know, 17 and whatever, 364 days, and tomorrow 18, and then they just treated like a completely different human being or an individual. And there is no support.*”

One parent shared her dismay when told that her daughter’s provider would no longer be able to provide services. This parent (Parent 9) was told, “…*oops, we were actually supposed to cut her off at 16, but okay, we’ll do one more, but then you’re going to have to find someone else for her. And I’m like, wait, what now? When was someone going to tell me that?* Another parent stated, *“Yeah, but it’s just so ironic too this week when I got your email and I had just gotten a letter from our [insurance company] saying they weren’t going to cover the pediatrician anymore*”.

### 3.2. Theme Two: Transfer of Care Barriers

Parents cited a myriad of barriers they encountered as their sons’ or daughters’ care was transferred from the pediatric to adult-focused system of health care. These barriers involved challenges in locating new adult-focused primary and specialty health care providers, being able to access services in a timely manner, and obtaining needed medical supplies and durable medical equipment. Throughout the discourses evident in the subthemes, parents experienced frustration, distress, and worries about their children’s health status and access to care. The major theme, *Transfer of Care Barriers,* consists of the following nine subthemes: *Difficulty in Finding Providers/Waitlists*; *Difficulty Finding Primary Care and Specialty Providers; Difficulty Finding Mental Health Providers*; *Difficulty in Finding Dentists*; *Changes in Insurance; Discrimination; and Difficulty Obtaining Medical Supplies or Equipment.*

#### 3.2.1. Subtheme One: Difficulty in Finding Providers/Waitlists

Many parents spoke about the transfer of care barriers associated with finding providers. The challenges in locating providers extended beyond identifying available providers. Parents discovered that some providers imposed service access restrictions based on their sons’ or daughters’ insurance coverage (i.e., Medicaid). Other parents encountered providers who expressed discomfort with providing care due to their limited clinical experiences with serving this population. Some of their comments reflecting these issues were as follows: *“We haven’t transferred, we haven’t found any anybody”* (Parent 1); “*By the insurance, there is lot of limitations in terms where to go and there is no plan*” (Parent 6). “*A lot of folks are like, oh, I don’t know what [INDISTINCT] [LAUGHS] I can’t help you. So, that process of finding someone new took a while”* (Parent 9). One parent (Parent 6) summarized her process of trying to locate a provider: “*So I don’t know, it’s just been a frustrating process. I don’t know how to make it easier or what the solutions are, but it’s just been frustrating*.” Another parent (Parent 8) detailed the prolonged process undertaken to locate a provider that reflected her cultural preference in the selection of a provider for her son. As she described, she attributed the delays to the lengthy enrollment to being unable to access their provider of choice:

“*I called the insurance company, they told me I could look online, and so I did. I thought well, you know, let me find somebody. And I found, you know, because I’m Hispanic, I thought, well, maybe I’ll feel more comfortable. And so I went out of my way to look for somebody Hispanic. I looked (INDISTINCT) Hispanic name. And so I waited. And we got the card, and I was happy. And I called to make an appointment for my son for the initial appointment. Because it had been months already that we had been struggling to find a provider. And so when I called, they told me that the doctor no longer took Medi-Cal.*”

A few parents identified the challenges they encountered in trying to access providers, including being added to waitlists, which caused delays in accessing needed services. This issue is evident with the following parental comment (Parent 6): “*So first there was a waitlist apparently to even I guess sign up for it. Then once you’ve signed up for it, then it’s another month or so before you can even be seen by someone. So it’s just been sort of this I don’t think we were really prepared.*”

#### 3.2.2. Subtheme Two: Difficulty Finding Primary Care and Specialty Providers

Parents repeatedly shared the difficulties they encountered with finding adult-focused primary and specialty care providers. As the parents reported, they were largely left on their own to locate providers, whether it was for primary or specialty care. Several parents referred to the problems in locating a primary care provider (PCP). It was apparent that parents received scant assistance in locating health care providers for the transfer of care. Finding a PCP was a challenge for many of these parents, as demonstrated by the comments. As Parent 8 noted, “*Very little was helpful to me. I struggled through the whole process, and it took a long time for us to find a provider.”* One parent (Parent 1) noted, *We’re not able to find the right PCP for….”*. Another parent (Parent 6) remarked on the following response when attempting to contact PCP office/clinic: *“And some, you’re either listed somewhere and then all of a sudden you call them and they’re like, oh no, sorry we’re not taking any more new patients or we’re not taking any.*”

Two parents revealed the barriers they encountered in accessing needed specialty care. Parent 11 revealed the problems faced in trying to access physical therapy services for her son:

“I had to take him to physical therapy. And this place, the first day I went, they said, no, we can’t take him because he has special needs, he needs to go back to **** or he needs to back to ***. But the insurance, it keeps doing this.”

Parent 6 referred to the ongoing problems in locating an ophthalmologist for her son. She recounted the need to continually change physicians who could effectively provide services to her son: “*I’ve been having the same problem to eye doctors. Now I got a doctor because I changed probably about four doctors. They don’t know how to work with a special needs. And they treat my son like a normal person.*”

#### 3.2.3. Subtheme Three: Difficulty Finding Mental Health Providers

Two parents cited problems with accessing mental health services for their children. For these parents, trying to access these needed services for their children and the scarcity of services available was particularly frustrating. One parent (Parent 4) stated, *“I have problems, mainly with mental care… there’s absolutely nothing. There’s not a single program that help the parents. There is not a single resource help the parents.”* Another parent (Parent 5) referred to the significant changes encountered with the current access and level of mental health services her son now receives that occurred as a result of moving from another state, illustrating how service differences vary by state:

“*And so it took me quite a bit of time for me to actually find the extra help that I actually needed for my son. Because I would have therapists come out to my house like three times a week in Las Vegas to where I don’t have that here.*”

#### 3.2.4. Subtheme Four: Difficulty with Finding Dental Providers

This parent’s input reflected the scope of frustration she experienced, as well as the challenges other parents encountered: “*But it just seems like anybody we talk to, especially dental, it’s so hard. Nobody knows how to work with my son. Everywhere I go, I have to fight.*” (Parent 8). Parent 11 shared the challenges she encountered in trying to find a dentist for her son: “*It was difficult to find a dentist. It took me two years because the other dentist where he used to go before were not specialized in special needs, so they won’t see him.*”

#### 3.2.5. Subtheme Five: Changes in Insurance

Several parents identified problems with accessing adult-focused providers due to having to change their health insurance plans. During the transfer of care, parents discovered the extent of changes they faced with their children’s insurance coverage that they had not anticipated. This transfer of care barrier was exemplified by the following comments: “*The insurance does not cover them…*” (Parent 4); “*And recently, our insurance situation changed and we had to go on Covered California*” (Parent 6). Another parent (Parent 10) expressed frustration with the lack of informational support received concerning insurance coverage for her son:

“*But you know, now he’s 26. So no, the process is complicated. Nobody explains, and least of all the doctors or the medical officers. They at least now I think there is some kind of a system of maybe navigator, but back then, no. And so without having the private insurance, I would have been lost.*”

#### 3.2.6. Subtheme Six: Discrimination

As above, multiple parents expressed difficulty in finding providers who would care for their son or daughter with a disability; however, some of the statements expressed a degree of discrimination in offices or a lack of accessibility in offices. One parent (Parent 8) shared their experience of discrimination: “I can’t believe that even now people are still, like this technician, you know, the MRI technician was like, well, look at him, you know.” Another parent (Parent 9) stated, “*And so, it happened. I found out, found a place where she could go. And that was after a few missed attempts of taking her to places and looking around going, this is not the place for her. They don’t have any of the equipment that she needs. [LAUGHS] And they’re looking at me like, what do you want us to do with her?”*

#### 3.2.7. Subtheme Seven: Difficulty Obtaining Medical Supplies or Equipment

For parents, obtaining supplies became more difficult after the transfer of care to adult-focused providers occurred. Several comments made by parents reflect this dilemma: “*He’s having incontinence issues now so I didn’t know you could get diapers, pull-ups from …. until another parent tells you. Because I’ll tell the social worker what’s going on, but they won’t offer a lot*” (Parent 1). Parent 8 reflected her frustration with trying to access services: “*So I’d have to call again, called and called. And I never got a response*”. 

### 3.3. Theme Three: Transfer of Care Facilitators

Parents identified a number of positive experiences associated with their sons’ or daughters’ transfer of care. Eight subthemes were generated that depicted the array of facilitators that assisted parents and their sons or daughters during the transfer of care. The six subthemes associated with Theme Three are Finding Med-Peds Doctors; Able to Stay with Current Provider; Parent Networking; Parent Advocacy; Cultural Congruency; and Benefit of Switching to Adult Provider/New Set of Eyes.

#### 3.3.1. Subtheme One: Finding Med-Peds Doctors

There were a variety of parental perspectives offered about the feasibility of locating a meds-peds physician (i.e., a physician whose specialty practice includes both pediatrics and internal medicine) for their son or daughter. Several parents shared positive comments about accessing care from a med-peds doctor/lifespan provider, although they themselves had not actually accessed a meds-peds physician. Parent 7 remarked about the unavailability of meds-peds doctors but has been able to make the adjustments needed for her son’s care, as evidenced in these remarks:

“*I haven’t really run into any. [Son J]’s pretty healthy, you know, as far as all the problems he could have. I mean he’s got thyroid issues, so he has to have his blood drawn. But that’s been pretty stable. And like adult onset psoriasis, and so we’re working with the dermatologist. But the whole family has seen this dermatologist and [Son J]’s the youngest, and she had no problem taking care of [Son J]. So that was good.*”

As Parent 9 noted, she gathered information about accessing a meds-peds doctor in an atypical manner: “*And so, to that point, at a conference, one of the presentations about your child getting older, they had mentioned, hey, a [Med-Ped] doctor, when it’s time to make that transition, if you can find a Med-Ped doctor, that’s kind of ideal, because they’re a little bit more trained in what our kids need. And I mentioned that to her neurologist at the time, and they knew of a clinic that did that, had that. And I eventually was able to find somebody. But like I said, had I not known to use that wording, I’d probably still be looking for somebody.*“

#### 3.3.2. Subtheme Two: Able to Stay with Current Provider

Several parents who were interviewed expressed relief that their sons or daughters could continue to receive care either for a longer period of time or that the provider’s continuity of care would continue into adulthood. Their stress reprieve was evident in their statements. Of all the parents interviewed, one parent indicated that her daughter was provided care until she was 21. She noted (Parent 8), “*Actually, we had a wonderful pediatrician, so she was very helpful. I guess that was the part that was helpful. She was able to keep us on till the age of 21. So, she stretched it out as much as she could.*” One of the parents (Parent 3) shared the following information about her son’s provider that was atypical compared to the information shared by other parents: “Well you know, as far as the healthcare realm, for my son things didn’t really change. There hasn’t been a difference as far as provider because he had been with *****, so he’s just been continuing on. He’s been with **** I think since age 14 or so. He was no longer with a pediatrician at that point.”

#### 3.3.3. Subtheme Three: Parent Networking

Several parents spoke about the benefits of networking with other parents. One parent (Parent 10) noted, “*…speak to other parents who’ve already gone through it. It’s more helpful than speaking to health providers, or even the insurance company.*” *Another parent (Parent 7) shared that* “*…the best thing that I found was just networking with other parents with Down Syndrome.*” Parent 8 described the helpfulness of networking with other parents: “*And I talked to parents. A lot of parents are having the same issues that I did. Like oh, no, you know? Even a woman I talked to, she said she had changed doctors three times. Because she was trying to get IHSS for her daughter. And none of the doctors want to sign forms for her to be able to get IHSS. So nobody really had, you know, and I actually started talking to parents who had older kids [LAUGHS]. And so one of them at least gave me a good lead for a dentist.*”

#### 3.3.4. Subtheme Four: Parent Advocacy

A few parents cited their own advocacy actions on behalf of their children. In all cases, these parents were effective. Parent 8 shared the conversation she had with the provider, wherein she advocated for him to be seen: “*So I was really complaining. And I said I’m going to file a complaint…We’ve been waiting months to get you guys…And so they did call me back, like after a couple of hours. And they took him, you know. That was nice.*”

Parent 9 spoke about the advocacy activities she was involved with, not only for her daughter, but also for the disability community: “*I’m on a committee right now where we’re trying to develop something, at least for our community. Now, if other folks want to use it, then great. [LAUGHS] But so many of us are having this, and it’s across the board. There’s people on this community from all over, from Florida to California, New York to whatever. Because we’re all dealing with it. It may look different because of insurance, or whatever the case may be. But it’s the same problem all over.*”

One of the interviewed parents spoke about the relevance and importance of listening to parents. This parent (Parent 9) shared, “*And so far, in my experience in selection of most doctors, not all, the doctors that are willing to listen to me have actually been able to assist my son. And those doctors who feel I know my s stuff and you’re just a mother, his problems have not been solved.”*

#### 3.3.5. Subtheme Five: Cultural Congruency

A parent (Parent 10) of Indian ethnicity noted that the selection of a physician of the same ethnicity was important. She noted, “*…I selected a doctor whose name sound of my ethnicity. I’m Indian. …I mean he is from India and very traditional….so it worked out he said I know nothing about autism, so I’m going to your opinion on it and I’m going to listen to you. And that was the best thing happened to me.*” Another parent (Parent 8) stated this intent but then ran into the issue of the doctor no longer taking patients: “*I called the insurance company, they told me I could look online, and so I did. I thought well, you know, let me find somebody. And I found, you know, because I’m Hispanic, I thought, well, maybe I’ll feel more comfortable. And so I went out of my way to look for somebody Hispanic. I looked (for a) Hispanic name. And so I waited. And we got the card, and I was happy. And I called to make an appointment for my son for the initial appointment. Because it had been months already that we had been struggling to find a provider. And so when I called, they told me that the doctor no longer took Medi-Cal.*

#### 3.3.6. Subtheme Six: Benefit of Switching to Adult Provider/New Set of Eyes

One of the parents shared extensive thoughts about the benefits of accessing adult-focused health care providers. This parent (Parent 9) discovered that having a new set of providers resulted in renewed clinical considerations of benefits in relation to the care of her daughter:

“*Her pediatric GI decided to keep her. Her dentist kept her. But you find out, in time, that there becomes even issues with that. You don’t have the issue of needing to find a new doctor, but you run into things that are.. Because while they were, at least in my case, I’ve told her many times, you’re going to still be my baby when you’re 99 [LAUGHS]. But they’re not babies. And so, there’s those adult issues that can pop up, that the pediatrician isn’t going to know to look for, hasn’t dealt with. There’s medications You, your child, your child’s doctors have all known them for some period of time, it’s like when someone gets their haircut,” or loses weight, or whatever, if you see them every day, initially you may go, something’s different, I don’t know. But if it’s somebody that’s seeing them for the first time, or hasn’t seen them in a while, they’re going to notice quicker than someone that’s looking at them every day, and it’s just kind of become, you are who you are, it is what it is [LAUGHS].*”

### 3.4. Theme Four: Transfer of Care Recommendations

Parents offered a number of recommendations for improving the transfer of care to adult-focused care. These recommendations were based upon their lived experiences of the transfer of care to adult-focused providers. Theme Four is composed of the following subthemes: Parental Planning Ideas to Facilitate the Transfer of Care; Pediatrician Referral to Adult Primary Care Provider (PCP); Accessing Resources and Parental Planning Ideas; Parental Navigation Would Be Helpful.

#### 3.4.1. Subtheme One: Parental Planning Ideas to Facilitate the Transfer of Care

Many parents offered recommendations for facilitating the transfer of care to adult-focused providers. Each of the clusters of recommendations was based upon the parents’ personal experiences, much of which was based on hindsight. One parent (Parent 9) offered the following recommendation to assist parents with the transfer of care. She suggested that parents should be asking the following straightforward question of their pediatrician: “*How long can you see my child, so that I can prepare for that.*” *[LAUGHS].* Two parents suggested that starting early in planning the transfer of care is appropriate. As one parent (Parent 11) shared, “*…by the time they transition, they already should have referrals about these doctors online …*”. Parent 9 offered a similar perspective: “*Just so you know, this is what’s going to happen in a couple of years, you might want to start looking into that.*” Another parent (Parent 8) thought having a list of providers would be helpful for parents. This parent remarked, “I needed to know doctors who are friendly towards people with disabilities. And I wish there was such a list.” One parent (Parent 1) thought her child would benefit from staying as long as possible with the pediatric provider. She offered the following recommendation: “ *… just stay with your pediatrician as long as they’ll let you.*” Several parents offered a number of recommendations for successful planning of the transfer of care. Parent 2 recommended to “*Have good communication with doctors and plan ahead with the doctors.* Another parent (Parent 9) recommended “*….having something, somewhere, that parents can refer to that A, helps them understand the process.*”

#### 3.4.2. Subtheme Two: Pediatrician Referral to Adult Primary Care Provider (PCP)

Two parents thought that having the pediatrician suggest a referral to an adult-focused provider would be helpful. One parent (Parent 11) offered this strong opinion in this regard: “*So I think it’s an obligation from the pediatrician doctor to refer to the adult doctors.”* Parent 4 shared the assistance she received for accessing an adult-focused neurologist for her son:” *I have a doctor, a neurologist, and she gave me a recommendation of who might be enabled for that. So those recommendations and the fact that they have to be taking in account with the doctor when the transition comes to place.”*

#### 3.4.3. Subtheme Three: Accessing Training Resources

One of the parents (Parent 8) cited, as a recommendation, accessing resources. This parent stated, “*I wish even if like, you know, ….somebody would have like trainings. Or a website where I could go and get good resources for transitioning.*” Many parents offered several recommendations to improve the transfer of care. One parent (Parent 8) suggested the following: “*And talk about disabilities, and more awareness. And invite parents to the trainings and things.*” Another parent (Parent 10) made the following recommendation: “*But maybe, you know, just for that sake, maybe join forums and ask about adult doctors.*”

#### 3.4.4. Subtheme Four: Parental Navigation Would Be Helpful

A few parents offered recommendations that accessing parental navigation with the transfer of care would be helpful. As one parent (Parent 8) observed, “*It’s usually more parents, you know. (LAUGHS) Because we’re the ones looking for the resources to help our children, you know, a caregiver or a parent.*” Parent 9 echoed similar sentiments: “*So, if there was a specific program where that kind of peer support is offered, or mentorship, or whatever you want to call it, I would think a lot of parents would definitely welcome that.*”

## 4. Discussion

As evident in this study, the eleven parents who were interviewed offered perspectives about their health care transition journey with their sons or daughters with IDD. These parents were able to enumerate both a wide array of barriers and facilitators that impacted not only their sons’ or daughters’ HCT but also their personal experiences as parents who served as their children’s sources of support and advocacy throughout this process. These parents shared a range of emotions, as evidenced in their descriptions of the barriers and the facilitators they faced during their sons’ or daughters’ HCT. As revealed by these parents, the barriers they encountered far outnumbered the facilitators.

Most notable and prevalent in the barrier themes and subthemes generated from these interviews were the difficulties parents encountered in their efforts to find health and dental care services for their sons or daughters. The barriers parents encountered were not only relegated to finding an adult-focused primary or specialty provider with experience and expertise in IDD but also to other barriers that compounded the transfer of care. These additional barriers were associated with insurance issues, service capacity limits, necessitating waitlists and service delays, and parents’ perceptions of discrimination, as their sons or daughters would be turned away from health services due to their disability. These findings are supported by previously reported investigations [27,28,29,30,31,32,33].

Other studies have reported providers’ lack of comfort with the provision of services to adults with childhood-acquired chronic conditions and IDD. This barrier has been reported widely in the HCT literature [27,28,29,30,31,32]. Provider hesitancy and resistance in providing services to individuals with IDD have been reported as attributable, in part, to workforce issues, as medical, nursing, and allied health programs have limited didactic and clinical training in the provision of services to IDD populations [33]. Given the lack of workforce capacity, service delays are an inevitable outcome for the IDD population, as has been reported extensively in the literature.

In this study, parents reported their needs for assistance with navigating the health care system for adults. Their familiarity and confidence with managing their sons’ or daughters’ care in the pediatric system of care were tested with the transfer of care to the adult-focused system of care. As these parents reported, they were ill-prepared by pediatric providers to navigate the new challenges they faced in accessing adult-focused care. These parents provided rich descriptions of the barriers they faced, which were pervasive when trying to access primary, specialty, and mental health care for their sons or daughters and have been previously reported in other studies [19,26,27,31,33].

The interviewed parents offered a number of facilitators that were helpful during the HCT process. Parents described transfer of care strategies that created more optimal timing for accessing adult-focused providers either earlier or later than the projected date of exit from pediatric care. Other parents searched for meds-peds physicians after learning about this specialty group, a specialty group not widely reported in the parent literature or on resource websites.

Foremost among their perspectives was the assistance and support they received from other parents [19]. This assistance was given in the form of informational resources obtained through networking with other parents. Parental support groups, such as Family Voices, family resource centers, and parent-to-parent networks, are generally known by parents and accessed for support and services.

This group of parents offered a variety of recommendations based upon their own lived experiences with their sons’ or daughters’ HCT. These suggestions intersect with the facilitators they identified, such as accessing resources and including parents who can assist other parents navigate the new system of care as members of the service team [19,26]. They advised starting early to avoid the transfer of care glitches that parents have described (i.e., insurance issues) and to be an informed consumer and advocate for their children during this period of time [34]. Parents recommended that pediatric providers be more involved with the process by providing provider referrals or a list of providers that they could seek out, which they felt was lacking in many cases.

This study brings to light many areas of potential improvement in the HCT process. Perhaps increased provider training about caring for adults with IDD, as well as the legal responsibilities to provide accessible care, as stated in the ADA, could assist with this process. Various efforts to improve provider education for the care of individuals with IDD exist [35], but these efforts must continue to be strengthened and disseminated. In addition, the concepts of pediatric providers being able to refer to adult providers is an important one; however, this may be challenging in practice when pediatricians may not necessarily know of adult providers who are able to take the patients’ insurance, or, for the reasons stated previously, are not willing to care for their patients with IDD as they reach adulthood. Furthermore, it may be important to look at payment systems to ensure that providers are able to take extra time if needed to care for adults with IDD and to have access to case management to assist with issues such as durable medical equipment, as parents stated this was a challenge to obtain in adult-serving systems [20,27,33].

### Limitations

There are several limitations associated with the study findings. Firstly, all data were collected anonymously, thereby limiting the full understanding of the social determinants of health that may be associated with the perspectives and experiences of these parents and their sons or daughters. The limited collection of demographic data can decrease the generalizability of a study. However, this study could be replicated in additional settings with the collection of additional demographic information. In this sample, just one parent requested Spanish interpretation, which is not representative of the minority–majority status that exists in California. Also, despite the identification of barriers that parents identified, these families live in a state where those who qualify for developmental disabilities services are enrolled in an entitlement system of care through regional centers. Parents whose children receive entitlement services may have different lived experiences pertaining to service provision. That is, parents in this study, compared to other states, are likely to have more affordable (no cost/low cost) IDD service and support options regardless of family income levels. Only a few states in the United States have IDD entitlement programs. Given the aforementioned service model, the findings of this small qualitative sample may not be representative of the HCT needs identified in other IDD populations. Another study limitation that must be considered is respondent bias; that is, the parents may not fully disclose their perceptions of their sons’ or daughters’ health care transition experience. It may be that parental recall of their experiences is hampered by memory recall, in that relevant experiences are forgotten or mischaracterized. It may be that parents who participated in this study were influenced by the interviewer herself regarding her nonverbal behaviors or interview style, or the interview process itself may have affected parental responses. Parents who participated in this study may have been influenced by social desirability, responding to questions posed by the interviewer that would appear to be more desirable responses rather than expressing their more candid reflections. Lastly, selection bias needs to be considered. It is unknown the extent to which parents who consented to participate in the study are representative of those who choose not to participate.

Of note, the Framework Method was utilized in this study as it is highly systematic and can be useful in interdisciplinary groups and/or with researchers with varying degrees of qualitative research experience^31^; however, it is not a method that is useful in all qualitative studies, particularly those that involve life-history analysis or analyzing recorded conversations between patients and their healthcare providers [29].

## 5. Conclusions

Eleven parents of sons or daughters with IDD offered their perspectives about the HCT experience they had been involved in with their children. Analysis of their interviews revealed four major themes and subthemes that reflect the barriers and facilitators they encountered during this process. In addition, parents offered recommendations that would be useful for others. The major themes generated from the analysis of parental interviews consisted of two major themes that were related to HCT barriers: Pediatric Care Contrasted with Adult-Focused Care and Transfer of Care Barriers. There were also two major themes pertaining to HCT facilitators: Transfer of Care Facilitators and Transfer of Care Recommendations. The major themes and their subthemes provide insights and more informed understandings of the commonalities that the parents of sons or daughters with childhood-acquired conditions experience and the unique challenges they face. These findings provide additional support for the results reported in other HCT studies of the lived experiences of parents and their sons or daughters during the transition period. These findings will inform clinicians of service-related practices and programs that this population of youth and young adults with IDD may require to achieve optimal HCT outcomes. These findings will call to policymakers’ attention the shortcomings of the transition service delivery model that need to be addressed to improve HCT outcomes effectively.

## Figures and Tables

**Table 1:** Parent demographics.

Parent	Gender of Adult Child	IDD Diagnosis	Parent/Legal Guardian	Interview Language Used
1	Male	Not Disclosed	Parent	English
2	Female	Not Disclosed	Parent	English
3	Male	Not Disclosed	Parent	English
4	Male	Down Syndrome	Parent	English
5	Male	Not Disclosed	Parent	English
6	Male	Not Disclosed	Parent	English
7	Male	Epilepsy	Parent	Spanish
8	Male	Autism	Parent	English
9	Male	Autism	Parent	English
10	Female	Autism	Parent	English
11	Male	Down Syndrome	Parent	English

## Data Availability

Data are not available due to privacy restrictions.
